# Data-Driven Based Approach to Aid Parkinson’s Disease Diagnosis

**DOI:** 10.3390/s19020242

**Published:** 2019-01-10

**Authors:** Nicolas Khoury, Ferhat Attal, Yacine Amirat, Latifa Oukhellou, Samer Mohammed

**Affiliations:** 1 Laboratory of Images, Signals and Intelligent Systems (LISSI), University of Paris-Est Créteil (UPEC), 122 rue Paul Armangot, 94400 Vitry-Sur-Seine, France; nicolas.khoury@u-pec.fr (N.K.); amirat@u-pec.fr (Y.A.); samer.mohammed@u-pec.fr (S.M.); 2 French Institute of Science and Technology for Transport, Development and Networks (IFSTTAR), University of Paris-Est, COSYS, GRETTIA, F-77447 Marne la Vallée, France; latifa.oukhellou@ifsttar.fr

**Keywords:** Parkinson diseases, gait cycle, wearable sensors, vertical ground reaction forces (vGRFs), features selection method, classification

## Abstract

This article presents a machine learning methodology for diagnosing Parkinson’s disease (PD) based on the use of vertical Ground Reaction Forces (vGRFs) data collected from the gait cycle. A classification engine assigns subjects to healthy or Parkinsonian classes. The diagnosis process involves four steps: data pre-processing, feature extraction and selection, data classification and performance evaluation. The selected features are used as inputs of each classifier. Feature selection is achieved through a wrapper approach established using the random forest algorithm. The proposed methodology uses both supervised classification methods including K-nearest neighbour (K-NN), decision tree (DT), random forest (RF), Naïve Bayes (NB), support vector machine (SVM) and unsupervised classification methods such as K-means and the Gaussian mixture model (GMM). To evaluate the effectiveness of the proposed methodology, an online dataset collected within three different studies is used. This data set includes vGRF measurements collected from eight force sensors placed under each foot of the subjects. Ninety-three patients suffering from Parkinson’s disease and 72 healthy subjects participated in the experiments. The obtained performances are compared with respect to various metrics including accuracy, precision, recall and F-measure. The classification performance evaluation is performed using the leave-one-out cross validation. The results demonstrate the ability of the proposed methodology to accurately differentiate between PD subjects and healthy subjects. For the purpose of validation, the proposed methodology is also evaluated with an additional dataset including subjects with neurodegenerative diseases (Amyotrophic Lateral Sclerosis (ALS) and Huntington’s disease (HD)). The obtained results show the effectiveness of the proposed methodology to discriminate PD subjects from subjects with other neurodegenerative diseases with a relatively high accuracy.

## 1. Introduction

Parkinson’s disease (PD) is a slow, progressive, chronic neurodegenerative disorder. It is the second most common neurological disease (after Alzheimer’s disease) and affects an enormous portion of the elderly population worldwide [1,2,3]. Globally, nearly 5 million people are affected by this disease [4]; however, that number could double by 2030. In France, 200,000 people [5] suffer from Parkinson’s disease, and approximately 25,000 new cases [6] are diagnosed each year. The average age of diagnosis is 58 years [7], but 20% are under 50 years old at diagnosis. However, rare genetic forms (approximately 5%) can lead to an early occurrence before the age of 40 [8,9,10]. The disease begins 5 to 10 years before any clinical symptoms appear, at which point approximately half of the dopaminergic neurons have disappeared. The existence of various non-typical signs such as depression, pain, fatigue, etc. can render the diagnosis more difficult. Each patient presents specific characteristics: disease evolution is unique to each person and depends on many factors. Some symptoms such as difficulty speaking can appear after several years or may remain insignificant.

Currently, the diagnosis made by doctors to assess the severity level of PD is conducted using various methods based on several research domains, including cognitive deficits [11,12,13,14,15,16,17], speech disorders [18,19,20], human stability [21,22], gait cycle and others. It is clear that the lack of specific test for Parkinson’s disease makes it challenging to diagnose PD subjects. In addition, signs and symptoms similar to those of Parkinson’s disease may have other causes, such as dementia with Lewy bodies, progressive supranuclear palsy, and certain types of stroke. Similar symptoms can also be observed in the case of exposure to some toxins, intake of some antipsychotic medication, as well as a head injury [23]. This might further complicate the PD diagnosis using only qualitative criteria such as bradykinesi, hypertonia, depression, pain, fatigue, etc. Hence, machine learning based tools have shown recently great interest to assist physical doctors in their daily diagnosis process. Therefore, the main objective of this work is to provide a useful machine learning tool that aims at supporting physiotherapists in the diagnosis process as well as providing appropriate analysis and selection of relevant clinical features extracted from the subject’s vertical Ground Reaction Forces (vGRFs) during walking.

**Figure 1:**
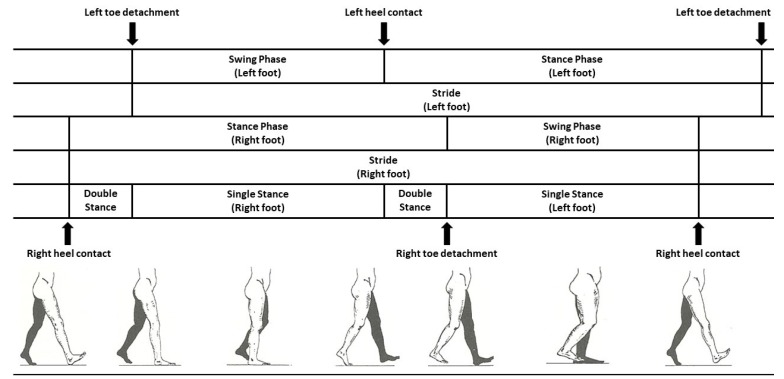
Gait-phase representation; swing, stance and double stance phases for both feet [26].

This study exploits gait cycle analysis to diagnose PD. Gait disturbances can often be observed as PD progresses. Among these disturbances, such as halting gait, festinating gait and short steps can simplify the diagnosis of PD. Furthermore, the study of gait parameters such as step length, step frequency and velocity [24] is useful in understanding the mechanism of human motor control and in recognising neurological disease progression [25]. In this context, the study of the gait cycle has been strongly applied to evaluate gait pattern disorders. A single gait cycle starts when the toe of the right foot (RFo) and the heel of the left foot (LFo) are contacting the ground simultaneously, and it ends with the same configuration. Figure 1 shows the composite of human gait cycle.

This article presents a review of different classification methods used to discriminate between healthy subjects and PD. The classification uses vertical Ground Reaction Forces (vGRFs) data collected from the gait cycle. An online dataset collected within three different studies is used in this study. The PD diagnosis process includes four steps: data pre-processing, feature extraction and selection, data classification and performance evaluation. The selected features are used as the input to each classifier. Feature selection is achieved through a wrapper approach established using the random forest algorithm. The proposed methodology can use both supervised classification methods such as K-nearest neighbour (K-NN), decision tree (DT), random forest (RF), Naïve Bayes (NB), support vector machine (SVM) and unsupervised classification methods such as K-means and the Gaussian mixture model (GMM). In this study, the performances of these methods are compared with respect to different metrics: classification rate (accuracy), precision, recall and F-measure. Furthermore, a gait dataset of neurodegenerative diseases patients including patients suffering from Amyotrophic Lateral Sclerosis (ALS), also called Lou Gehrig’s disease, and Huntington’s disease (HD), is used to evaluate the effectiveness of the methodology to accurately recognize PD affected subjects among subjects suffering from neurodegenerative diseases. The generalization performance evaluation is carried out using the leave-one-out cross-validation method. Here, the primary objective for implementing these classification techniques is to review, compare and evaluate their performances. Clinical-based features are extracted from vGRFs collected from gait cycles. First, the classification methods undergo a selection phase that chooses the most relevant features combination; then, the resulting features are used as inputs to each classifier. The rest of this paper is organized as follows. Section 2 is dedicated to related works. Section 3 presents a description of the datasets used in the study. Section 4 presents the methodological context of the study—in particular, the classification techniques and performance evaluation. Section 5 presents the experimental results and a discussion, Finally, Section 6 provides conclusions and perspectives for future work.

## 2. Related Works

The effects of PD on the evolution of the stride-to-stride variability during a gait cycle have been extensively studied in the literature [27]. Yogev et al. [28] studied the cognitive function and the effects of different types of dual tasks on the gait of subjects with PD. Their results show that the executive function [29,30,31] had deteriorated in the subjects with PD. In [32], the authors discuss gait asymmetry (GA) in subjects with PD. The outcomes of this study show that when gait becomes impaired and less automatic, Gait Asymmetry apparently is linked to cognitive inputs and dual tasks. In the same context, Hausdorff et al. [33] focused on the gait dynamics to evaluate the effect of Rhythmic Auditory Stimulation (RAS), which consists of using musical stimuli to enhance the gait performance of neurological conditions subjects (e.g., subjects with PD). It is shown that RAS promotes more automatic movement and reduces stride-to-stride variability in subjects with PD. The study conducted in [34] showed that the ability to maintain a steady gait with low stride-to-stride variability decreases in subjects with PD. In [35], the authors showed that swing time variability is independent of gait speed in subjects with PD; therefore, it can be used as a marker of rhythmicity and gait steadiness. The results show an increase in the variability of stride time and swing time at comfortable walking speeds for the subjects with PD compared to control subjects.

To analyse the gait cycle, two types of sensors are generally used: wearable [36,37,38,39,40,41,42,43,44,45,46,47] and non-wearable [47,48,49,50,51,52,53,54,55,56,57]. From the literature, using wearable sensors to enable gait analysis is a convenient, efficient and low-cost approach that has been adopted to provide helpful information in several health-related applications [58]. In [36], Jean et al. studied the classification of spatial-temporal image of plantar pressure (STIP) using an in-shoe dynamic foot pressure system. Mariani et al. [46] presented a method based on on-shoe wearable sensors and a processing algorithm to provide measurements that characterize the mobility symptoms of PD during Timed Up and Go (TUG) and gait tests. The following spatio-temporal parameters were used in the study: swing width, turning, path length and inter-cycle variability.

Classification of patients with PD has been extensively studied based on the use of ground reaction force sensors placed in shoes [59,60,61]. The vGRFs measurements were used most often in previous studies. For example, the PD classification in [59] is based on vGRFs and uses a simple threshold-based classifier. However, this method has disadvantages because of its sensitivity to the choice and tuning of the threshold values [62]. In [59], Su et al. introduced measures of gait asymmetry by comparing the ground reaction force (GRF) features of both the left and right limbs. The effectiveness of the proposed measures was evaluated by differentiating between the walking patterns of patients with Parkinson’s and healthy subjects, respectively. The differentiation was done through threshold-based and Multi-Layer Perceptron (MLP) models. A classical cross validation procedure has been used to estimate the classifier performances, involving the dataset being randomly divided into three subsets that are: training (80%), validation (10%) and test (10%) subsets.

Machine learning based approaches to classify patients with PD can be divided into two learning approaches: supervised and unsupervised [63]. In [60], the authors proposed classifying patients with PD and healthy control subjects using gait analysis through deterministic learning theory. This classification approach consists of two phases: a training phase and a classification phase. In the classification phase, a bank of dynamic estimators was constructed from all the training data. The results show that this approach achieves an accuracy rate of 96.39%. In [64], vGRFs obtained from idiopathic subjects with PD were used to extract wavelet-based features, which, in turn, were used as neural network inputs that used weighted fuzzy membership functions to classify subjects with PD. In [65], extracted features from gait signal measurements acquired through eight ground-reaction force sensors placed underneath each foot, and SVM-based algorithm were used to classify 93 subjects with PD and 73 healthy control subjects. The results show that the proposed approach achieved an accuracy of 91.20% at diagnosing the subjects with PD. In [61], SVM-based algorithm and extracted/selected feature form time series-based information such as stride intervals, swing intervals’ measurements acquired through force-sensitive resistor sensors were used to PD diagnosis. The classification accuracy for patients with PD was approximately 89.33%. For PD diagnosis, the stride interval density and its sub-phases (swing and stance intervals) were estimated using the non-parametric Parzen-window method and least squares SVM (LS-SVM) was used in [66]. The obtained classification rate was approximately 90.32%.

Khorasani et al. [25] used a hidden Markov model (HMM) with Gaussian mixtures to classify patients with PD and healthy subjects. The proposed method allows achieving an accuracy of 90.3%. In [67], to classify subjects with PD and healthy subjects, stride signal variance, regression error, mean and variance of phase signal and Petrosian dimension features and nearest-mean scaled classifier were used. This approach resulted in a classification accuracy of 95.6%. In [68], IMU gait measurement sequences sampled during walking are encoded as hidden Markov models (HMMs) to extract their dynamics. The distance between HMMs is learned and employed in a standard nearest neighbour classifier. This approach achieved an accuracy of 85.51%. A Q-back propagated time delay neural network classifier was proposed in [69], which builds a temporal classification model to monitor and to predict the severity of gait disturbances in subjects with PD by analysing the instability in their walking patterns. The dataset used includes data from three PD research studies [70]. The results show that the classification accuracy on the three sub-datasets reached 91.49%, 92.19% and 90.91%, respectively. In [71], Ertugrul et al. proposed an approach built using shifted one-dimensional local binary patterns and machine learning. The statistical features extracted as: energy, skewness, correlation, coefficient of variation, entropy and kurtosis. These features were classified using Naïve Bayes, multilayer perceptron, partial C4.5 decision tree, random forest, Bayes Network, logistic regression, a rule learner method and functional tree methods. The best accuracy rate obtained was 88.88% by the multilayer perceptron classifier. In [72], an RF algorithm was used for classification, and a set of features in the time and frequency domains were extracted. The classification accuracy when all features subsets were used reached 98.04%.

Joshi et al. [73] presented an approach that combined wavelet analysis and an SVM to distinguish Parkinson’s subjects from healthy ones using gait cycle variability. The results showed that adopting the wavelet transform approach resulted in a classification rate of 90.32%. In [74], the parameters of approximate entropy, normalized symbolic entropy, and signal turn counts were computed to measure stride fluctuations in patients with PD. To implement gait pattern classification, Wu et al. employed generalized linear regression analysis and an SVM. The experimental results showed that the SVM achieved an accuracy of 84.48%. In [75], several supervised classifier methods including SVM, RF, K-NN and DT were compared in terms of the classification performances. Furthermore, this study compared different kernel functions, including linear, Gaussian, quadratic and cubic. The results show that the SVM with the cubic kernel outperformed the other classifiers and achieved an accuracy of 93.6%. In [76], an approach exploiting the principle of the repetition of gait cycle patterns was used to discriminate healthy subjects from PD subjects. To evaluate the gait cycle repetition, a Continuous Dynamic Time Warping (CDTW) technique was proposed. The CDTW distances extracted from vGRF signals corresponding to stance phases are used as inputs of several classifiers to differentiate healthy subjects from PD. This approach achieved an accuracy rate ranging within 80.02–97.5%. In [77], linear discriminant analysis and K-means were used to classify and cluster subjects with PD and healthy control subjects. The goal of the authors was to study the effect of neurodegenerative diseases (i.e., Parkinson’s disease) on mobility and gait in comparison with healthy control subjects. In [78], K-means was used with the objective of discriminating patients with PD from control subjects. Finally, in [25,79], Parkinson’s disease diagnosis was made based on gait recognition using GMM. Table 1 presents a synthetic review of studies on PD diagnosis.

The most of aforementioned studies are mainly based on the use of time-domain and frequency-domain features to diagnosis Parkinson’s disease. However, such features could not be easily linked to a clinical indicator. In this paper, the main objective is to develop a useful tool to aid the diagnosis of Parkinson’s disease using clinical-based features extracted from vertical Ground Reaction Forces (vGRFs). This tool is mainly devoted to being used in a clinical environment to support physiotherapists in the PD diagnosis process. Hence, in this study, only clinical-based features are considered.

## 3. Dataset Description

**Figure 2:**
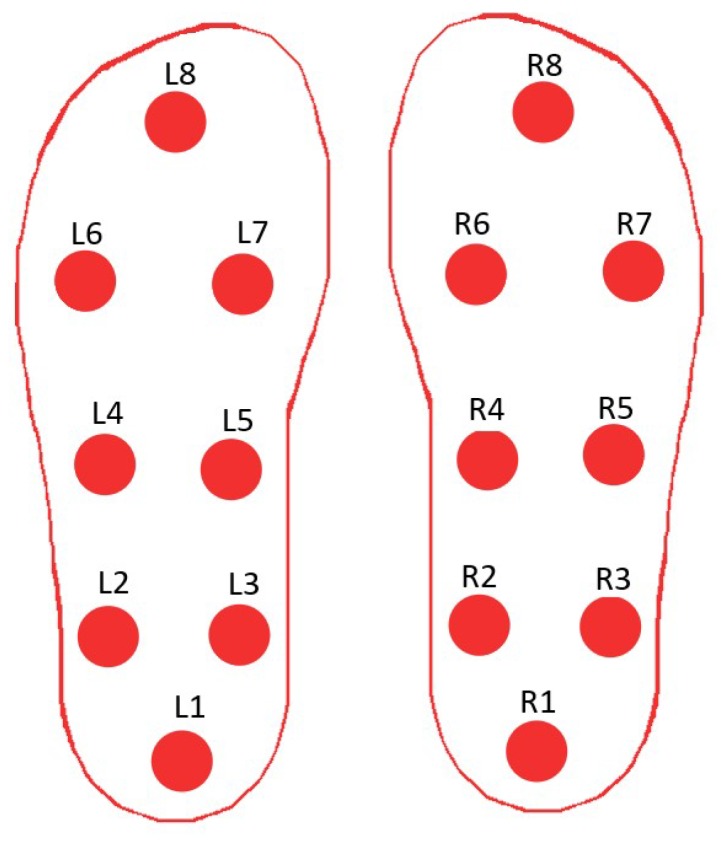
Placement of the 16 sensors under both feet.

**Figure 3:**
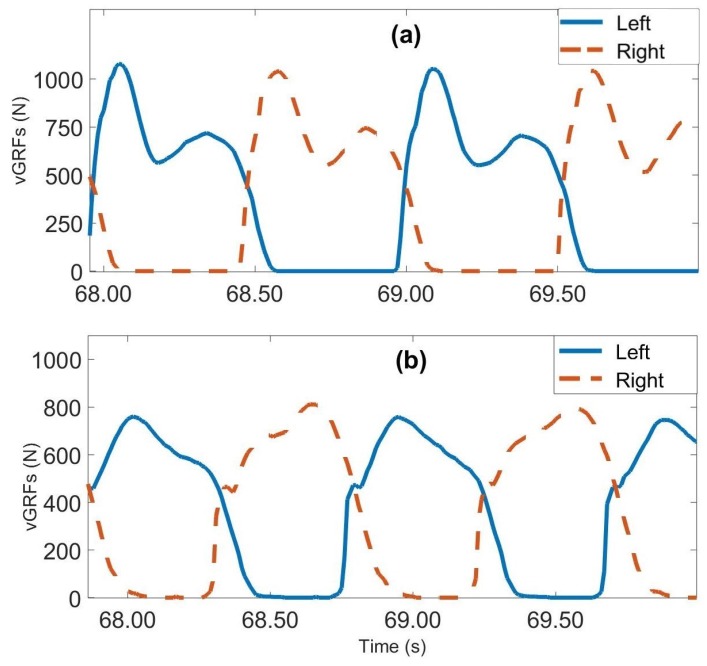
vGRFs measured on left foot (blue) and right foot (red); (**a**) healthy subject, (**b**) subject with PD.

The gait dataset used in this study was obtained from the PhysioNet web site [70]. It contains gait data for 93 patients suffering from Parkinson’s disease and 72 healthy subjects. The average age of both categories is approximately 66 years. Males constitute 63% of the subjects with PD and 55% of the healthy subjects. This dataset contains three different sub-datasets. The first one, provided by Yogev et al. [28], contains the gait data of 29 people with PD and 18 healthy subjects. The second, provided by Hausdorff et al. [33], includes the gait data of 29 people with PD and 25 healthy individuals. The third, provided by Frenkel-Toledo et al. [80], contains the gait data of 35 people with PD and 29 healthy people. Each dataset includes vGRF measurements collected from eight force sensors (Ultraflex Computer DynoGraphy, Infotronic Inc., Hong Kong, China) placed under each foot of the subjects as shown in Figure 2. The vGRF signals are sampled at a frequency of 100 Hz. To create the different sub-datasets, the participants were asked to walk at their typical walking pace on level ground for periods ranging from 2–5 min for distances ranging from 25 m to 77 m. These three studies differ in their measurement protocols. Subjects in [28] were asked to walk under different dual tasking conditions. Subjects in [33], were asked to walk with and without Rhythmic auditory stimulation (RAS) condition, while, in study [80], subjects were asked to walk with and without assistance by using a wheeled walker on a motorized treadmill. It should be noticed that the subjects who participated in the three studies [28,33,80] were either healthy subjects or suffering from PD, excluding any other walking pathologies. In addition to the sixteen signals provided by the vGRFs sensors, the dataset also includes two signals that represent the sums of the eight sensor outputs for each foot. Demographic information, measures of disease severity on the Hoehn and Yahr (H and Y) scale, the Unified Parkinson’s Disease Rating Scale (UPDRS) and the Time Up and Go test (TUAG) are also included in this sub-dataset. Figure 3 presents the sum of the signals from the eight left and eight right superposed sensors of two subjects, one healthy and one diseased.

## 4. Background on Data Processing and Classification Techniques

This section presents the steps involved in Parkinson’s Disease classification.

### 4.1. Data Pre-Processing

**Figure 4:**
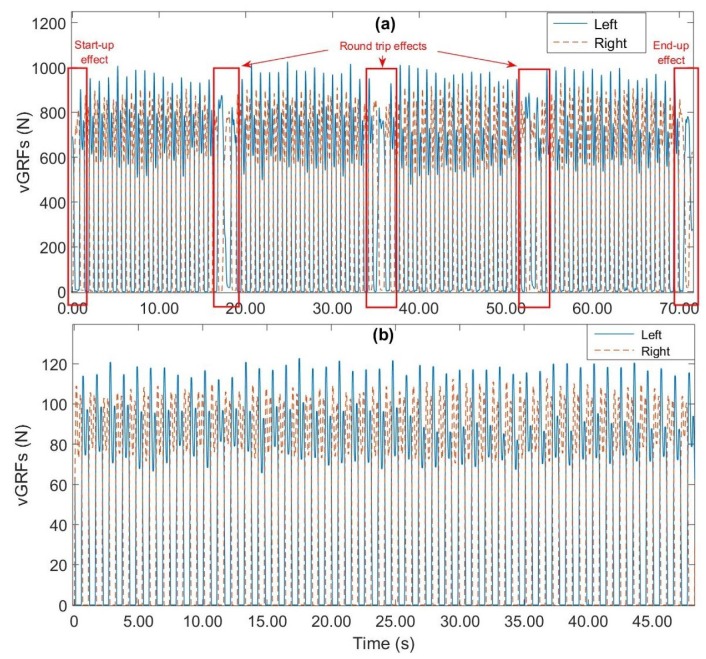
Example of vGRF data pre-processing; (**a**) raw vGRFs data; (**b**) processed vGRF data.

In this study, the two signals representing the sums of the eight sensor outputs from each foot are used. Rather than using the each individual sensor signal, these two signals allow stance and swing phase detection at high precision. Moreover, these two signals can reflect the overall conditions of fluctuation from one aspect of gait dynamics [60]. As described in [33], the subjects were asked to perform a round trip along a walkway; this pattern may reveal the presence of outliers in gait parameters. The recorded gait data during the turn-around phase were removed manually. In addition to the turn, the first and last 20 s were removed to ignore starting and stopping effects. An analysis of the collected vGRFs reveals some signal fluctuations. These phenomena are more important during the swing phase and lead to non-zero vGRFs values. To address this issue, a 10-point median filter is applied. Figure 4 shows the results after pre-processing the vGRF signals for one of the subjects.

### 4.2. Feature Extraction and Selection

#### 4.2.1. Feature Extraction

In this study, the most relevant spatiotemporal features from the clinical point of view are extracted from vGRF signals [28,32,34,35,81,82]. A total of nineteen features were extracted; these features are summarized in Table 2.

#### 4.2.2. Feature Selection

Feature selection is an important step before applying classification algorithms because it improves the overall classification performances and reduces the complexity and computation time of the algorithms. To achieve accurate classification results, using more than one feature is common. However, increasing the number of features may lead to the so-called curse of dimensionality, which results in a degraded classifier performance and increases the computation time and model complexity. Therefore, to increase the discriminant power of a classification algorithm, feature selection is required. The feature selection step involves finding the most relevant subset of features from the original feature set by eliminating inappropriate or redundant features.

Formally, feature selection can be described as follows: given a feature set $Q=\{q_{1},q_{2},...,q_{n}\}$ of size *n*, where *n* is the total number of features, and an evaluation function $E_{val}$, the purpose of feature selection is to find the optimal subset $Q^{\prime }$, where $\left(Q^{\prime }\subset Q\right)$ and has a size of $n^{\prime }$, where $\left(n^{\prime }<n\right)$ such that the following equality is verified:

(1) $$E_{val}\left(Q^{\prime }\right)=\underset{M\subset Q}{\mathrm{argmin}}E_{val}\left(M\right),$$

where $∥M∥=n^{\prime }$, and $n^{\prime }$ is a number predefined by the user or controlled by the criterion used in the selection process. The feature selection process can be subdivided into three phases: (1) generation of subsets *M* containing $n^{\prime }$ candidates from the original set using various strategies; (2) evaluation of the generated subsets using various evaluation models (during this phase, some candidates are added or discarded from the selected feature set); and (3) halting the feature selection process using specific termination criteria [83]. The generation phase can be achieved using exhaustive, heuristic or random searches. An exhaustive search can find the most relevant subset; however, it may be extremely time-consuming. The heuristic and random search methods attempt to reduce the computational complexity by compromising the performance. Several authors categorize the evaluation phase into three categories, namely, filter methods [84], wrapper methods [85] and hybrid methods [86]. The filter methods operate directly on the set of features and weight or rank each feature. These methods exploit the intrinsic feature properties and no classifier is involved. Unlike the filter methods, the wrapper methods use a classification step to evaluate the selected features based on their predictive accuracy. These methods often yield better results than the filter methods do. Finally, the hybrid methods use the internal parameters of the classification algorithms to select the most relevant subset of features. Regarding the last phase, a stopping criterion is generally determined to terminate the feature-selection process. Several criteria can be used—for example, a minimum number of features, a maximum number of iterations, a good classification rate, a maximum calculation time, and so on [87].

### 4.3. Classification Techniques

In this section, the classification techniques used in this study, namely, K-NN, DT, RF, SVM, NB, GMM and K-means, are briefly described.

- K-nearest neighbour [88] is one of the simplest supervised classification approaches. It is a non-parametric supervised classification method. In K-NN, no explicit or modelling phase occurs before the classification phase. Classification with K-NN involves two main steps: (1) a distance calculation (usually, Euclidean distance) is made between the new sample and all training samples; (2) the new sample is assigned to the majority class of the nearest samples using the K nearest neighbour selection.
- The support vector machine is a well-known supervised machine learning model [89] that is used primarily for binary prediction problems. The underlying idea of this model is based on the concepts of a hyper-plane and the margin. The learning process consists of finding a linear separator (also called a hyperplane) which separates the training data while maximizing the margin between the hyperplane and these training data. In some cases, SVM cannot directly find a linear separation between the data in its original representation. Thus, to be able to find a linear separator between the groups, a training data transformation proposed by Vapnik [89] is performed from the original space to another higher dimensional space. This transformation can be made using a kernel function such as the Gaussian, quadratic, or polynomial kernel functions.
- The decision tree is a supervised classification method [90] that is simple, effective and easy to interpret. A DT finds nonlinear relationships between the inputs and outputs of the system. A DT is an iterative classifier that separates variables into branches and nodes. The nodes are composed of one root node and diverse inertial nodes and leaves. Several algorithms have been used for DT construction including the Classification and Regression Tree (CART) [90], Iterative Dichotomiser (ID3) [91] and C4.5 [92], etc.
- The random forest is another supervised machine learning introduced by Breiman in [93]. As its name implies, a random forest is constructed from a set of DTs. Each tree is constructed using a training subset generated randomly from the original dataset using the Bootstrap technique. Therefore, the RF model combines the bagging technique and the randomized selection from partitioning the data nodes during DT construction.
- Naïve Bayes (NB) is another simple supervised machine learning model based on the Bayes theorem [94,95] with independence assumptions between observation data. NB’s main advantage is that its learning model is simple and does not require any complicated iterative parameter estimation. Despite its simplicity, the NB model can outperform more sophisticated machine learning models.
- The Gaussian Mixture Model is a supervised and unsupervised probabilistic machine learning model. This model represents the training data as weighted-sum finite Gaussian-component densities. The data are represented according to one or multi-Gaussian distributions and characterized by the covariance matrix and the mean vector. The parameter estimation for this model (the proportions, the covariance matrices of the Gaussian component and the mean vectors) is based on the maximization of the log likelihood using the expectation–maximization (EM) [96] algorithm.
- K-means is still another simple unsupervised machine learning model. This method divides the training data into *k* homogeneous clusters [97]. The objective is to minimize the total intra-cluster variance and the distortion measure as a cost function. The K-means model finds the cluster centroids iteratively and assigns the data to the various cluster centroids based on their distance (e.g., Euclidean) until convergence occurs.

### 4.4. Performance Evaluation

To evaluate the performances of the different classification techniques, accuracy, precision, recall and F-measure are used as performance metrics. These metrics are defined as follows.

The proportion of correctly classified samples is measured by the accuracy metric. For binary classification, accuracy is calculated as follows:

(2) $$\mathrm{Accuracy}=\frac{T_{p}+T_{n}}{T_{p}+T_{n}+F_{p}+F_{n}}.$$

where the true positives ($T_{p}$), true negatives ($T_{n}$), false positives ($F_{p}$) and false negatives ($F_{n}$) respectively represent the correct assignment of positive examples, the correct assignment of negative examples, the incorrect assignment of positive examples into the negative classes and the incorrect assignment of negative examples into the positive classes. In addition, the following metrics are also used to evaluate the performances of the proposed approach.

- The Precision metric measures the proportion of relevant subjects that are relevant. It measures the ability of the classifier to refuse irrelevant subjects. The Recall metric evaluates the proportion of relevant subjects that are found. It measures the ability of the classifier to provide all relevant subjects. These metrics are expressed as follows:
  (3) $$\mathrm{Precision}=\frac{T_{p}}{T_{p}+F_{p}}.$$
  (4) $$\mathrm{Recall}=\frac{T_{p}}{T_{p}+F_{n}}.$$
- The F-measure metric is a combination of precision and recall defined as follows:
  (5) $$F-\mathrm{measure}=\frac{(1+\beta^{2}).Precision.Recall}{\beta^{2}.Precision+Recall}.$$
  where β is a real positive weighting factor, used to set the degree of importance of the precision and recall. In this study, β is set to 1 to assign the same weight to both precision and recall.

### 4.5. Parameters Setting

Each classification technique requires one or several parameters that control (affect) the prediction outcome of the classifier. Choosing the best values for these parameters is difficult and involves finding a trade-off between the model’s complexity and its generalization ability. In this study, finding parameter settings is conducted using a grid search. A grid search consists of adapting a grid of values (2D or 3D, depending on the number of model parameters) and incrementing each parameter by a fixed interval until the optimal values of the parameters are found. For example, for a model with two parameters *a* and *b*, the procedure consists of varying parameter *a* with a predefined interval $\left[a_{min},a_{max}\right]$ using an increment of ▵a and parameter *b* in the interval $\left[b_{min},b_{max}\right]$ with an increment of ▵b. For each vector of values (a,b), the model’s performance in terms of recognition rate is evaluated, and the vector that yields the best accuracy is selected. The advantage of this method is that it allows the optimal parameters within the chosen intervals to be selected, but it is expensive in terms of computation time.

The selected parameters for each model are described below:

#### 4.5.1. Supervised Algorithms

- A K-NN with Euclidean distance is applied to the three sub-datasets (Yogev, Hausdorff, and Frenkel-Toledo). The number of neighbours is determined by varying K from 2 to 10. The optimal K values for the Yogev, Hausdorff and Frenkel-Toledo sub-datasets are, respectively, 7, 2 and 7.
- The CART algorithm is used for the DT model. The CART uses the Gini index (Gini impurity) parameter to find the best construction and the best partition of the tree.
- For the RF model, the number of trees is varied between 10 and 200. The optimal numbers of trees for the Yogev, Hausdorff and Frenkel-Toledo sub-datasets are, respectively, 100, 80 and 150.
- For the NB model, a normal distribution is used to model the conditional probability of the observation data and classes for the three sub-datasets.
- For the SVM model, a nonlinear model with polynomial kernel function (degree 3) is used for the two first sub-datasets, and a linear model is used for the third sub-dataset.

#### 4.5.2. Unsupervised Algorithms

- For the GMM model, the diagonal Gaussian function is used for the Frenkel-Toledo sub-dataset, and the full Gaussian function is used for the other two sub-datasets.
- For K-means, the only parameter to tune is the number of classes, which in this study is two (subjects with PD and healthy subjects).

After the parameter-setting step, each sub-dataset is divided into training and testing sets according to the leave-one-out cross-validation procedure. For the supervised approaches, the labels are used during the learning phase; then, during the testing phase, the labels estimated by each classifier are matched with the reference labels (true labels) to compute the classification performances. Unlike the supervised models, the unsupervised models are trained using only the extracted features; no reference labels are used; instead, the labels are used only for classification evaluation purposes. Note that (1) all extracted features and (2) just the selected features are used as classifier input. To select the most relevant features, each sub-dataset is considered separately. Random forest features selection method [90] including 100 trees features is used. Each node in the decision trees considers a single feature to split the dataset based on a given optimal condition using the Gini impurity measure. Thus, the impurity decrease from each feature is calculated for each tree. Then the impurity decrease measure from each feature is averaged, and the features are ranked according to this measure. Thus RF allows reordering the extracted features according to their relevance degree (percentage). In this study, a set of five features representing 80% of the cumulative relevance is selected for each sub-dataset.

## 5. Results and Discussion

The classification techniques described above were implemented to classify subjects with PD. Five supervised classification techniques, namely, K-nearest neighbour (K-NN), SVM, DT (CART), RF and NB, as well as two unsupervised techniques, the GMM and K-means, were compared using standard evaluation metrics. The inputs to both the supervised and unsupervised approaches are the extracted and selected features from the raw data.

### 5.1. Parkinson’s Disease Classification Results

In this subsection, the performances of the different classification techniques are presented and discussed. Note that, in this study, the three sub-datasets are analysed separately.

Table 3 shows the obtained results in terms of accuracy for the different classifiers with and without feature selection. This table presents the obtained accuracy from the three sub-datasets (Yogev, Hausdorff and Frenkel-Toledo). Table 3 also shows that using only the features obtained from the feature selection method as classifier inputs leads to improving the overall accuracy rate with respect to the case where all the extracted features were used. For the Yogev sub-dataset, improvements of approximately 3%, 3%, 1%, 2%, 3%, 10% and 3% can be observed when using K-NN, CART, RF, NB, SVM, K-means and GMM, respectively. Almost the same improvements can be observed for the Hausdorff. (2%, 1%, 1%, 5%, 2%, 4% and 6%, respectively) and Frenkel-Toledo (1%, 3%, 4%, 4%, 1%, 3% and 8%, respectively) sub-datasets. This outcome can be explained by the fact that the feature selection method, by providing the best combination of relevant features for the classification algorithm, improves the classification performances with respect to both healthy subjects and PD subjects. Moreover, by analyzing the performances of each classifier with and without feature selection, it can be noted that, in the case of K-NN, CART and NB with selected features, an improvement of 3% for Yogev’s sub-dataset is observed compared to the results obtained when using all extracted features. In the case of NB with selected features, an improvement of 5% can be noticed for the Hausdorff’s sub-dataset. For the Frenkel-Toledo sub-dataset, an improvement of 4% in the case of CART, NB and SVM can be observed. The remaining cases of the classifiers allow for an improvement ranging from 1 to 2% for the three sub-datasets. However, a greater improvement can be observed in the case of the Hausdorff sub-dataset (5%). Regarding the unsupervised classifiers, K-means shows an improvement of 3% in the case of Hausdorff and Frenkel-Toledo sub-datasets. Regarding the Yogev sub-dataset, using K-means with selected features allows achieving a significant improvement about 10%. A significant improvement can be also noted in the case of GMM when using Hausdorff and Frenkel-Toledo. Finally, there is also a slight improvement in the case of the Yogev sub-dataset. GMM using selected features achieves an improvement about 3%. These results show that the selected features can further increase the discriminative capability of the different classifiers (supervised and unsupervised). It is worth noting that using selected features as classifier inputs allows not only the improvement of the classification performances but also a significant reduction of the computational time both in the training and testing steps.

Table 4 summarizes the results of the feature selection method by presenting the five selected features for each sub-dataset. In the following sections, we use references to the features listed in Table 4 as substitutes for their names. These features are used as the input of each classifier. The features referred to as 6, 7 and 13 are included in the feature combination obtained from the Yogev and Hausdorff sub-datasets. We found that this combination was the most effective in achieving the highest correct classification rate for these two sub-datasets. A combination of the features referred to as 7 and 11 are obtained from the first and third sub-datasets. Moreover, the combination of the features referred to as 7, 8 and 19 (derived from the former two) yields good performances on the Frenkel-Toledo sub-dataset.

Table 5, Table 6 and Table 7 show the classifier performances in terms of accuracy, precision, recall and F-measure when using the selected features with the Yogev, Hausdorff and Frenkel-Toledo sub-datasets. A comparison of the classifier performances shows that RF, K-NN and SVM achieve almost similar accuracy while outperforming the other classifiers with the Yogev sub-dataset. The same observation can be made when considering the precision, recall and F-measure metrics. K-means and GMM exhibit the worst rate performances in terms of accuracy, precision, recall and F-measure. On the Frenkel-Toledo sub-dataset, RF, SVM, CART and RF have almost similar accuracy, precision, recall and F-measure outperforming the other classifiers. Again, K-means and GMM exhibit the worst performances. Finally, on the Hausdorff sub-dataset, K-NN achieves the highest accuracy, followed by SVM, RF, CART and NB. Regarding the precision metric, RF and SVM achieve almost similar performance, followed by K-NN, CART, and NB. In terms of recall and F-measure, K-NN classifier provides the best rates. The worst precision, recall and F-measure rates were obtained in the case of K-means and GMM. By comparing supervised and unsupervised methods, it can be noted that supervised ones outperform unsupervised methods.

By analyzing the result differences observed between the different sub-datasets, it can be noticed that almost all classifiers (supervised and unsupervised) show their best results in the case of the Hausdorff et al. sub-dataset. It can be also noticed that the results obtained in the case of Yogev et al. are better than those obtained for Frenkel-Toledo et al. This can be explained by the fact that the number of PD subjects with low severity are more important in the case of Frenkel-Toledo sub-dataset (28 among 35), followed by the Yogev et al. sub-dataset (15 among 29). Finally, the Hausdorff et al. sub-dataset includes the lower number of PD subjects with low severity (12 among 29). Unlike the number of PD subjects with low severity, the number of PD subjects with high severity is more important in the case of the Hausdorff sub-dataset (17 among 29) followed by the Yogev et al. sub-dataset (14 among 29). Finally, the the Frenkel-Toledo et al. sub-dataset includes the lower number of PD subjects with high severity (7 among 35). It is worth noting that the subjects with low severity (beginning stage of the disease) may be considered by the classifiers, in some cases, as healthy subjects. However, the PD subjects with high severity can be easily distinguished from healthy subjects.

To analyse the confusion that can occur in the classification step, global confusion matrices obtained using the different classifiers under the leave-one-out cross-validation on each sub-dataset (Yogev, Hausdorff and Frenkel-Toledo) are given in Table 8, Table 9 and Table 10. In most cases, the classifiers recognize subjects with PD better than they do with healthy subjects, particularly on the Yogev and Hausdorff sub-datasets. This can be explained by the fact that the number of healthy subjects in Yogev and Hausdorff sub-datasets is smaller than the number of subjects with PD (see Table 8 and Table 9). These subject imbalances may have affected the classifier performances because they cannot capture the specificities of the under-represented classes. On the Yogev sub-dataset (Table 8), the biggest percentage of misclassified healthy subjects is between 12% and 25%, which means that, among the 18 healthy subjects, four were classified as having PD. Most of the supervised methods classified 11 to 14% of subjects with PD as healthy, which means that, among the 29 subjects with PD, only four were misclassified. On the Hausdorff sub-dataset (Table 9), the percentage of misclassified healthy subjects in the case of K-NN, NB, and SVM varies from 16% to 40%, i.e., that, among 25 healthy subjects, 10 subjects were classified as having PD, whereas, in the case of supervised classifiers (except NB), 1 to 7% of subjects with PD were classified as healthy. This result means that, among the 29 subjects with PD, only two subjects were misclassified. In the Frenkel-Toledo sub-dataset, the number of healthy subjects is 29, whereas the number of PD ones is 35. Note that, in almost all cases, the healthy subjects are better recognized than are subjects with PD. Table 10 shows that the percentage of misclassified healthy subjects is between 13% and 24%, which means that, among the 29 healthy subjects, six subjects were classified as having PD. In contrast, 11 to 23% of subjects with PD were classified as healthy, which means that, among the 35 subjects with PD, eight subjects were misclassified. To explain this outcome, we observed that most of the subjects with PD who were misclassified as healthy subjects were in the beginning stage of the illness, according to the H & Y scale. The Yogev sub-dataset includes six subjects among 15 with a severity of 2 according to the H & Y scale, while the Hausdorff and Frenkel-Toledo sub-datasets include four and seven subjects among 12 and 28, respectively, with a severity of 2. Considering the misclassified healthy subjects, they were in the three sub-datasets mostly elderly, overweight, or had levels similar to that of PD on the TUAG test.

It is clear that comparing algorithm performance across different studies is a difficult task for many reasons. This difficulty is mainly related to: (i) the type of sensors used to quantify PD activities, (ii) the performance evaluation criteria (specificity, recall, precision, F-measure, accuracy, etc.), and (iii) the validation procedure (leave one out, P-fold, bootstrap). In this study, we have limited the comparison to the studies in literature considering the Physionet dataset. Table 11 summarizes the most relevant works for PD diagnosis using Physionet dataset. It can be noticed that almost all related studies use statistical features (time-domain and frequency-domain features) as classifier inputs. It can be also observed that the proposed approach outperforms major state-of-the-art performances. Certainly, using time-domain and frequency-domain features may lead, in certain studies, to a higher accuracy rate; however, such features could not be easily linked to a clinical indicator. As such systems are devoted to being used in a clinical environment to support doctors in the PD diagnosis process, it is necessary to use clinical-based features. Therefore, the main advantage of the proposed method is the use of only clinical-based gait features. On the other hand, we can notice a drawback related to the fact of not considering the family history and the medical history of the different subjects. Such information could lead to a more accurate PD diagnosis.

### 5.2. PD Discrimination from Other Neurodegenerative Diseases (Amyotrophic Lateral Sclerosis (ALS) and Huntington’s Disease (HD))

To further evaluate the effectiveness of the proposed methodology, we evaluated its performance in discriminating PD subjects from other patients suffering from other neurodegenerative diseases. For this purpose, an additional dataset collected from gait cycles of healthy, PD, Amyotrophic Lateral Sclerosis (ALS) and Huntington’s disease (HD) subjects is used [98]. This dataset contains gait features extracted from vGRFs data. 15 PD subjects, 20 HD subjects, 13 ALS subjects and 16 healthy subjects participated in the experiments. To generate the dataset, the participants were asked to walk at their typical walking pace along a 77m-long corridor for five minutes. A total of 12 gait features extracted from vGRFs data are provided. These features are combined to calculate the 19 features used in this study (see Section 4.2.1). To deal with the imbalanced data problem, the random under sampling method [99] is used.

Table 12, Table 13, Table 14 and Table 15 show the obtained results in terms of accuracy, precision, recall and F-measure in the binary classification between PD and ALS subjects, PD and HD subjects, PD and Healthy subjects, and PD and ALL (ALS, HD and Healthy) subjects, respectively. These results are obtained using five selected features, achieved through a wrapper approach based on the random forest algorithm. Moreover, the generalization performance evaluation is performed using the leave-one out cross validation. In the classification between PD and ALS subjects, K-NN provides the best performances rate, followed by SVM, RF, CART, NB, K-means and GMM. In the classification between PD and HD subjects, K-NN provides the best accuracy (83.33%), followed by SVM (80%), RF (76.67%), CART (73.33%), NB (70%), K-means (69.33%) and GMM (64.67%). The same observation can be made when considering the other performance metrics. In the PD and Healthy subjects classification, SVM provides the best accuracy rate (90.32%), followed by K-NN and RF with the same rate (87.10%), then NB (83.87%), CART (80.65%), GMM (65.16%) and finally K-means achieves the worst accuracy rate. By analyzing the other performance metrics, the same observation can be made. Finally, in the classification between PD and All subjects, K-NN achieves the best results in terms of accuracy precision, recall and F-measure with an accuracy rate of 90%, followed by SVM (86%), RF (83%), CART (80%) and NB (76.67%), then K-means (75%) and GMM (69%).

The obtained results show high accuracy rates in the discrimination between PD subjects and the remaining subjects (Healthy, ALS and HD subjects). This is due to the fact that each disease has its own impact on the gait pattern and can then be distinguished from other diseases. As described in [82,100], ALS subjects, compared to healthy ones, walk more slowly with longer average stride time, as well having a less steady and more temporally disorganized gait. Subjects with HD walk with shorter and more variable stride length, lower cadence, and greater variability in swing, stride and double support time. Finally, subjects with PD show a shortened stride length, increased stride-to-stride variability, reduced gait speed, freezing and shuffling gait.

It can be noticed that subjects with PD can be distinguished from subjects with ALS disease with a relatively high accuracy (92.86%). This result is due to the fact that PD and ALS subjects may share some common features in gait cycle. Consequently, PD subjects and ALS subjects display different gait rhythm, and the discrimination between them, based on gait analysis, can be done with a relatively high classification rate [101]. Considering the classification between PD and HD subjects, the accuracy rate is the lowest compared to the other cases of classification (83.33%), and this is due to the common effect of these two diseases on the gait pattern, which can display more similar gait rhythm [100,101]. In terms of classification between PD and Healthy subjects, the obtained results are approximately the same as those obtained with Hausdorff’s sub-dataset. Finally, in the classification between PD and All subjects (ALS, HD and healthy), the obtained results show that the proposed methodology allows differentiating between the PD subjects and the other ones with a very high accuracy rate (90%) when using the K-NN classifier.

## 6. Conclusions

In this study, we implemented several classification methods used to recognize PD based on vGRFs collected from gait cycles. This article discusses the complete structure of the PD recognition process: from data acquisition to performance evaluation. First, data acquisition and sensor placement are addressed. Then, feature extraction and selection processes are presented, followed by a theoretical background describing the various supervised and unsupervised classification methods. Finally, we presented a comparison of the five supervised methods (K-NN, CART, RF, NB and SVM) and two unsupervised methods (K-means and GMM). The five selected features were used as classifier inputs. The classifiers are compared in terms of classification rate (accuracy), precision, recall and F-measure. Their generalization performance is then assessed using the leave-one-out cross-validation. An additional gait dataset of neurodegenerative disease patients was used to confirm the effectiveness of the proposed methodology in discriminating PD subjects from subjects suffering from neurodegenerative diseases such as Amyotrophic Lateral Sclerosis (ALS) and Huntington’s disease (HD). The supervised classification approaches yield more efficient results, as it can be expected since they use labeled data in the learning phase. K-NN, RF and SVM provide good results in terms of accuracy and F-measure. The best accuracy results were obtained on the sub-dataset collected by Hausdorff. The main contribution of this paper is the use of three different sub-datasets, which make it possible to compare the various classification methods more completely. The principal points that make this work more generally, more effectively and more reliably are the extraction of a large number of features, utilization of the feature selection method and utilization of the classification methods belonging to both supervised and unsupervised approaches. The use of the second dataset confirmed that the proposed methodology is efficient for discriminating with a high accuracy PD subjects from ALS and HD subjects. Ongoing works concern the introduction of other features that may enhance the PD diagnosis. The information such the age, the weight and the Timed Up and Go test could be used to improve ability of the machine learning methods to discriminate Parkinson disease. Ongoing works concern also the handling of the imbalanced data problem. The cost-sensitive learning and ensemble methods are currently being investigated.

## Figures and Tables

**Table 1:** Synthetic review of studies on PD diagnosis.

References	Sensors	Sensors Type	Method	Validation Methods	Accuracy
Jean et al., 2008 [36]	In-shoe dynamic foot pressure	Wearable	SVM	15-fold cross validation	91.73%
Cho et al., 2009 [48]	CCD camera	Non wearable	MDC	Not specified	95.49%
Muniz et al., 2010 [56]	Force platform	Non wearable	LR, PNN, SVM	Bootstrap method	91–94%
Wu and Krishnan 2010 [66]	Force sensors	Wearable	LS-SVM	Leave-one-out	90.32%
Sarbaz et al., 2011 [67]	Force sensors	Wearable	Nearest mean scaled	70% (train), 30% (test)	95.6%
Daliri 2012 [61]	Force sensors	Wearable	SVM	50% (train), 50% (test)	89.33%
Lee et al., 2012 [64]	Force sensors	Wearable	NEWFM	50% (train), 50% (test)	74–77%
Daliri 2013 [65]	Force sensors	Wearable	SVM	50% (train), 50% (test)	84–91%
Khorasani et al., 2014 [25]	Force sensors	Wearable	HMM with GM	Leave-one-out	90.3%
Dror et al., 2014 [51]	Microsoft 3D camera sensor	Non wearable	SVM	leave-one-out	100%
Dyshel et al., 2015 [52]	Microsoft Kinect For Windows SDK	Non wearable	SVM	5-fold cross validation	-
Su et al., 2015 [59]	Force sensors	Wearable	Threshold-based	80% (train), 10%	72%
			and MLP models	(Valid.), 10% (test)	
Zeng et al., 2016 [60]	Force sensors	Wearable	RBF NN	5-fold cross-validation	96.39%
Jane et al., 2016 [69]	Force sensors	Wearable	Q-BTDNN	Cross-validation	90–92%
Ertugrul et al., 2016 [71]	Force sensors	Wearable	BayesNT, NB, LR, MLP,	10-folds	87–88%
			PART, Jrip, RF, and FT	cross-validation	
Cuzzolin et al., 2017 [68]	IMU sensors	Wearable	HMM	Cross-validation	85.51%
Açici et al., 2017 [72]	Force sensors	Wearable	RF	10-fold Cross Validation	74–98%
Joshi et al., 2017 [73]	Force sensors	Wearable	SVM	Leave one-out	90.32%
Wu et al., 2017 [74]	Force sensors	Wearable	SVM	Leave one-out	84.48%
Alam et al., 2017 [75]	Force sensors	Wearable	SVM, RF, K-NN, and DT	Leave one-out	85–95%
Bhoi et al., 2017 [77]	Force sensors	Wearable	K-means	-	-
Khoury et al., 2018 [76]	Force sensors	Wearable	K-NN, CART, RF, SVM, K-means and GMM	10-fold Cross Validation	80–97%
Aharonson et al., 2018 [78]	Force sensors and accelerometer	Non wearable	K-means	-	-
Haji Ghassemi et al., 2018 [79]	IMU sensors	wearable	GMM	-	-

**Table 2:** List of the nineteen extracted features.

Features References	Extracted Features	Explanation
1	Coefficients of Variations in percentage (%) of Swing Time of the left foot	$100\times \frac{Standard\;Deviation\;in\;percentage\;(\%)\;of\;the\;Swing\;Time\;of\;the\;left\;foot}{Mean\;in\;percentage\;(\%)\;of\;the\;Swing\;Time\;of\;the\;left\;foot}$ [28,34]
2	Coefficient of Variations in duration (s) of the Swing Time of the left foot	$100\times \frac{Standard\;Deviation\;in\;duration\;(s)\;of\;the\;Swing\;Time\;of\;the\;left\;foot}{Mean\;in\;duration\;(s)\;of\;the\;Swing\;Time\;of\;the\;left\;foot}$ [32]
3	Coefficient of Variations in duration (s) of the Stride Time of the left foot	$100\times \frac{Standard\;Deviation\;in\;duration\;(s)\;of\;the\;Stride\;Time\;of\;the\;left\;foot}{Mean\;in\;duration\;(s)\;of\;the\;Stride\;Time\;of\;the\;left\;foot}$ [28]
4	Coefficient of Variation in percentage (%) of the Swing Time of the right foot	$100\times \frac{Standard\;Deviation\;in\;percentage\;(\%)\;of\;the\;Swing\;Time\;of\;the\;right\;foot}{Mean\in \;percentage\;(\%)\;of\;the\;Swing\;Time\;of\;the\;right\;foot}$ [28,34]
5	Coefficient of Variation in duration (s) of the Swing Time of the right foot	$100\times \frac{Standard\;Deviation\;in\;duration\;(s)\;of\;the\;Swing\;Time\;of\;the\;right\;foot}{Mean\;in\;duration\;(s)\;of\;the\;Swing\;Time\;of\;the\;right\;foot}$ [32]
6	Coefficient of Variation in duration (s) of the Stride Time of the right foot	$100\times \frac{Standard\;Deviation\;in\;duration\;(s)\;of\;the\;Stride\;Time\;of\;the\;right\;foot}{Mean\;in\;duration\;(s)\;of\;the\;Stride\;Time\;of\;the\;right\;foot}$ [28]
7	Coefficient of Variation of the Short Swing Time	$100\times \frac{Standard\;Deviation\;of\;the\;Short\;Swing\;Time}{Mean\;of\;the\;Short\;Swing\;Time}$ [32]
8	Coefficient of Variation of the Long Swing Time	$100\times \frac{Standard\;Deviation\;of\;the\;Long\;Swing\;Time}{Mean\;of\;the\;Long\;Swing\;Time}$ [32]
9	Coefficient of Variation of the Gait Asymmetry	$100\times |ln\left(\frac{Coefficient\;of\;Variation\;of\;the\;Short\;Swing\;Time}{Coefficient\;of\;Variation\;of\;the\;Long\;Swing\;Time}\right)|$ [32]
10	Mean in percentage (%) of the Swing Time of the left foot	$100\times \frac{Duration\;(s)\;of\;Swing\;Time\;of\;the\;left\;foot}{Duration\;(s)\;of\;Stride\;Time\;of\;the\;left\;foot}$ (MeanofthePercentage(%)oftheSwingTimeoftheleftfoot) [28,34]
11	Mean in duration (s) of the Swing Time of the left foot	Timeoftheleftfootwasintheair (Meanoftheduration(s)oftheSwingTimeoftheleftfoot) [32,35,81]
12	Mean in duration (s) of the Stride Time of the left foot	TimefrominitialcontactoftheLeftfoottosubsequentcontactofthesamefoot (Meanoftheduration(s)oftheStrideTimeoftheleftfoot) [28,35,81,82]
13	Mean in percentage (%) of the Swing Time of the right foot	$100\times \frac{Duration\;(s)\;of\;Swing\;Time\;of\;the\;right\;foot}{Duration\;(s)\;of\;Stride\;Time\;of\;the\;right\;foot}$ (MeanofthePercentage(%)oftheSwingTimeoftherightfoot) [28,34]
14	Mean in duration (s) of the Swing Time of the right foot	Timeoftherightfootwasintheair (Meanoftheduration(s)oftheSwingTimeoftherightfoot) [32,35,81]
15	Mean in duration (s) of the Stride Time of the right foot	Timefrominitialcontactoftherightfoottosubsequentcontactofthesamefoot (Meanoftheduration(s)oftheStrideTimeoftherightfoot) [28,35,81,82]
16	Mean in percentage (%) of the Double Stance Time	$100\times \frac{Duration\;(s)\;of\;Double\;Stance\;Time}{Duration\;(s)\;of\;the\;Stride\;Time}$ (Meaninpercentage(%)oftheDoubleStanceTime) [34]
17	Mean of the Short Swing Time	compareSwingTimebetweentheRightandtheLeftfoot,thendistributitaccording totheShortSwingTimeCriteria(MeanoftheShortSwingTime) [32]
18	Mean of the Long Swing Time	compareSwingTimebetweentheRightandtheLeftfoot,thendistributitaccording totheLongSwingTimeCriteria(MeanoftheLongSwingTime) [32]
19	Mean of the Gait Asymmetry	$100\times |ln\left(\frac{Short\;Swing\;time}{Long\;Swing\;time}\right)|\left(averaged\;of\;the\;Gait\;Asymmetry\right)$ [32]

**Table 3:** Accuracy obtained with/without the use of the feature selection method, for each sub-dataset.

Sub-Dataset	Features Selection		Supervised					Unsupervised	
		Performances	K-NN	CART	RF	NB	SVM	K-Means	GMM
Yogev et al.	With	Accuracy	86.05%	83.72%	86.05%	74.42%	86.05%	63.72%	64.77%
	Without	Accuracy	82.56%	80.23%	84.88%	72.09%	82.56%	53.72%	61.39%
Hausdorff et al.	With	Accuracy	90.91%	84.30%	87.60%	77.69%	90.08%	55.12%	65.12%
	Without	Accuracy	88.43%	82.64%	85.95%	72.73%	87.60%	51.07%	58.93%
Frenkel-Toledo et al.	With	Accuracy	81.25%	79.69%	82.81%	79.69%	82.81%	57.19%	65.31%
	Without	Accuracy	79.69%	76.56%	78.12%	75%	81.25%	53.75%	57.34%

**Table 4:** The five most relevant features from each sub-dataset obtained using the RF feature selection method.

Sub-Datasets	References of Features	Selected Features
Yogev et al.	13	Mean in percentage (%) of the Swing Time of the right foot
	7	Coefficient of Variation of the Short Swing Time (SSWCV)
	10	Mean in percentage (%) of the Swing Time of the left foot
	6	Coefficient of Variation in duration (s) of the Stride Time of the right foot
	11	Mean in duration (s) of the Swing Time of the left foot
Hausdorff et al.	7	Coefficient of Variation of the Short Swing Time (SSWCV)
	5	Coefficient of Variation in duration (s) of the Swing Time of the right foot
	4	Coefficient of Variation in percentage (%) of the Swing Time of the right foot
	13	Mean in percentage (%) of the Swing Time of the right foot
	6	Coefficient of Variation in duration (s) of the Stride Time of the right foot
Frenkel-Toledo et al.	7	Coefficient of Variation of the Short Swing Time (SSWCV)
	19	Mean of the Gait Asymmetry
	11	Mean in duration (s) of the Swing Time of the left foot
	8	Coefficient of Variation of the Long Swing Time (LSWCV)
	9	Coefficient of Variation of the Gait Asymmetry

**Table 5:** Accuracy, Precision, Recall and F-measure for each classifier in the case of the Yogev et al. sub-dataset.

Performances	Supervised					Unsupervised	
	K-NN	CART	RF	NB	SVM	K-Means	GMM
Accuracy	86.05%	83.72%	86.05%	74.42%	86.05%	63.72%	64.77%
Precision	84.89%	82.86%	85.07%	74.73%	84.90%	64.34%	62.63%
Recall	86.34%	81.94%	85.07%	76.45%	85.71%	65.31%	62.91%
F-measure	85.61%	82.40%	85.07%	75.58%	85.30%	64.82%	62.77%

**Table 6:** Accuracy, Precision, Recall and F-measure for each classifier in the case of the Hausdorff et al. sub-dataset.

Performances	Supervised					Unsupervised	
	K-NN	CART	RF	NB	SVM	K-Means	GMM
Accuracy	90.91%	84.30%	87.60%	77.69%	90.08%	55.12%	65.12%
Precision	85.35%	82.34%	89.41%	70.64%	89.31%	52.58%	57.95%
Recall	88.35%	64.96%	71.48%	78.54%	78.96%	53.91%	61.29%
F-measure	86.83%	72.62%	79.45%	74.38%	83.82%	53.24%	59.57%

**Table 7:** Accuracy, Precision, Recall and F-measure for each classifier in the case of the Frenkel-Toledo et al. sub-dataset.

Performances	Supervised					Unsupervised	
	K-NN	CART	RF	NB	SVM	K-Means	GMM
Accuracy	81.25%	79.69%	82.81%	79.69%	82.81%	57.19%	65.31%
Precision	81.43%	79.56%	83.10%	79.69%	82.81%	61.07%	64.95%
Recall	81.67%	79.36%	82.22%	79.95%	83.10%	59.23%	64.62%
F-measure	81.55%	79.46%	82.65%	79.82%	82.96%	60.14%	64.78%

**Table 8:** Global confusion matrix obtained using the different classifiers in the case of the Yogev et al. sub-dataset.

		Obtained Classes													
		Supervised										Unsueprvised			
		K-NN		CART		RF		NB		SVM		K-Means		GMM	
		Healthy	PD	Healthy	PD	Healthy	PD	Healthy	PD	Healthy	PD	Healthy	PD	Healthy	PD
True	Healthy	87.50%	12.50%	75.00%	25.00%	81.25%	18.75%	84.38%	15.62%	84.38%	15.62%	71.53%	28.47%	55.63%	44.37%
Classes	PD	14.81%	85.19%	11.11%	88.89%	11.11%	88.89%	31.48%	68.52%	12.96%	87.04%	40.91%	59.09%	29.81%	70.19%

**Table 9:** Global confusion matrix obtained using the different classifiers in the case of the Hausdorff et al. sub-dataset.

		Obtained Classes													
		Supervised										Unsueprvised			
		K-NN		CART		RF		NB		SVM		K-Means		GMM	
		Healthy	PD	Healthy	PD	Healthy	PD	Healthy	PD	Healthy	PD	Healthy	PD	Healthy	PD
True	Healthy	84.00%	16.00%	32.00%	68.00%	44.00%	56.00%	80.00%	20.00%	60.00%	40.00%	51.84%	48.16%	54.76%	45.24%
Classes	PD	7.29%	92.71%	2.08%	97.92%	1.04%	98.96%	22.92%	77.08%	2.08%	97.92%	44.02%	55.98%	32.19%	67.81%

**Table 10:** Global confusion matrix obtained using the different classifiers in the case of the Frenkel-Toledo et al. sub-dataset.

		Obtained Classes													
		Supervised										Unsueprvised			
		K-NN		CART		RF		NB		SVM		K-Means		GMM	
		Healthy	PD	Healthy	PD	Healthy	PD	Healthy	PD	Healthy	PD	Healthy	PD	Healthy	PD
True	Healthy	86.21%	13.79%	75.86%	24.14%	75.86%	24.14%	82.76%	17.24%	86.21%	13.79%	80.97%	19.03%	57.24%	42.76%
Classes	PD	22.86%	77.14%	17.14%	82.86%	11.43%	88.57%	22.86%	77.14%	20.00%	80.00%	62.51%	37.49%	28.00%	72.00%

**Table 11:** Classification accuracy results obtained in recent related studies based on PhysioNet datasets.

Reference	Gait Parameters Features	Classifiers	Accuracy
Sarbaz et al., 2011 [67]	Time-domain features	Nearest mean scaled classifier	95.6%
Daliri 2012 [61]	Time domain features	SVM	89.33%
Lee et al., 2012 [64]	frequency domain features	NEWFM	74–77%
Daliri 2013 [65]	Time domain features	SVM	84–91%
Khorasani et al., 2014 [25]	Raw gait data	HMM with GM	90.3%
Ertugrul et al., 2016 [71]	Entropy, Energy, Correlation, Coefficient of Variation, Skewness and Kurtosis	BayesNT, NB, LR, MLP, PART, Jrip, RF, and FT	87–88%
Wu et al., 2017 [74]	ApEn, NSE, STC	SVM	84.48%
Jane et al., 2016 [69]	Left and right vGRF signals	Q-BTDNN	90–92%
Alam et al., 2017 [75]	Time and Frequency domain	SVM	85–95%
Açici et al., 2017 [72]	Time and Frequency domain	RF	74–98%
Khoury et al., 2018 [76]	CDTW-Distance	K-NN, CART, RF, SVM, K-means, and GMM	82–97%
Proposed methodology	Time domain features	K-NN, CART, RF, SVM, K-means, and GMM	80–91%

**Table 12:** Accuracy, Precision, Recall and F-measure for each classifier, obtained from the binary classification between PD and ALS subjects.

Performances	Supervised					Unsupervised	
	K-NN	CART	RF	NB	SVM	K-Means	GMM
Accuracy	92.86%	82.14%	85.71%	78.57%	89.29%	73.21%	66.79%
Precision	92.82%	82.14%	85.64%	78.46%	89.58%	77.89%	68.19%
Recall	92.82%	82.31%	85.64%	78.46%	88.97%	71.62%	65.46%
F-measure	92.82%	82.83%	85.64%	78.46%	89.28%	74.62%	66.88%

**Table 13:** Accuracy, Precision, Recall and F-measure for each classifier, obtained from the binary classification between PD and HD subjects.

Performances	Supervised					Unsupervised	
	K-NN	CART	RF	NB	SVM	K-Means	GMM
Accuracy	83.33%	73.33%	76.67%	70%	80 %	69.33%	64.67%
Precision	83.43%	73.76%	76.79%	70.83%	80.54 %	69.39%	64.88%
Recall	83.33%	73.33%	76.67%	70%	80%	69.33%	64.67%
F-measure	83.41%	73.54%	76.73%	70.41%	80.27%	69.36%	64.77%

**Table 14:** Accuracy, Precision, Recall and F-measure for each classifier, obtained from the binary classification between PD and Healthy subjects.

Performances	Supervised					Unsupervised	
	K-NN	CART	RF	NB	SVM	K-Means	GMM
Accuracy	87.10%	80.65%	87.10%	83.87%	90.32%	57.42%	65.16%
Precision	87.08%	80.63%	87.82%	85.31%	90.55%	58.52%	65.94%
Recall	87.08%	80.63%	86.88%	83.54%	90.21%	57.88%	64.73%
F-measure	87.08%	80.62%	87.35%	84.42%	90.38%	58.20%	65.33%

**Table 15:** Accuracy, Precision, Recall and F-measure for each classifier, obtained from the binary classification between PD and ALL (ALS, HD, and Healthy) subjects.

Performances	Supervised					Unsupervised	
	K-NN	CART	RF	NB	SVM	K-Means	GMM
Accuracy	90%	80%	83.33%	76.67%	86.67%	75.33%	69.33%
Precision	90.18%	80.54%	83.48%	76.79%	87.33%	76%	69.62%
Recall	90%	80%	83.33%	76.67%	86.67%	75.33%	69.33%
F-measure	90.09%	80.27%	83.41%	76.73%	87.00%	75.66%	69.47%
